# Improved Production of ε-Poly-L-Lysine in *Streptomyces albulus* Using Genome Shuffling and Its High-Yield Mechanism Analysis

**DOI:** 10.3389/fmicb.2022.923526

**Published:** 2022-05-31

**Authors:** Yongjuan Liu, Kaifang Wang, Long Pan, Xusheng Chen

**Affiliations:** ^1^Shandong Provincial Key Laboratory of Synthetic Biology, Qingdao Institute of Bioenergy and Bioprocess Technology, Chinese Academy of Sciences, Qingdao, China; ^2^The Key Laboratory of Industrial Biotechnology, Ministry of Education, School of Biotechnology, Jiangnan University, Wuxi, China; ^3^Shandong Energy Institute, Qingdao, China; ^4^Qingdao New Energy Shandong Laboratory, Qingdao, China; ^5^College of Biological Engineering, Henan University of Technology, Zhengzhou, China

**Keywords:** ε-poly-L-lysine, *Streptomyces albulus*, mutation breeding, genome shuffling, comparative genomics, high yield mechanism

## Abstract

ε-Poly-L-lysine (ε-PL), a natural food preservative, has recently gained interest and mainly produced by *Streptomyces albulus*. Lacking of efficient breeding methods limit ε-PL production improving, knockout byproducts and increase of main product flux strategies as a logical solution to increase yield. However, removing byproduct formation and improving main product synthesis has seen limited success due to the genetic background of ε-PL producing organism is not clear. To overcome this limitation, random mutagenesis continues to be the best way towards improving strains for ε-PL production. Recent advances in Illumina sequencing opened new avenues to understand improved strains. In this work, we used genome shuffling on strains obtained by ribosome engineering to generate a better ε-PL producing strain. The mutant strain SG-86 produced 144.7% more ε-PL than the parent strain M-Z18. Except that SG-86 displayed obvious differences in morphology and ATP compared to parent strain M-Z18. Using Illumina sequencing, we mapped the genomic changes leading to the improved phenotype. Sequencing two strains showed that the genome of the mutant strain was about 2.1 M less than that of the parent strain, including a large number of metabolic pathways, secondary metabolic gene clusters, and gene deletions. In addition, there are many SNPs (single nucleotide polymorphisms) and InDels (insertions and deletions) in the mutant strain. Based on the results of data analysis, a mechanism of ε-PL overproduction in *S. albulus* SG-86 was preliminarily proposed. This study is of great significance for improving the fermentation performance and providing theoretical guidance for the metabolic engineering construction of ε-PL producing strains.

## Introduction

ε-Poly-l-lysine (ε-PL) is a poly-cationic peptide with 25–35 L-lysine residues, and the peptide is connected through amide bond between α-carboxy group and ε-amino group (Najjar et al., 2007). ε-PL has antimicrobial activities against a wide spectrum of microorganisms including Gram-positive and Gram-negative bacteria, yeasts, and molds. It inhibits the growth of microorganisms by destroying the cell membrane structure, causing interruption of material, energy, and information transmission of cells, and ultimately leading to cell death (Melo et al., 2009; Ye et al., 2013; Santos et al., 2018; Zhang et al., 2018). Furthermore, ε-PL are water-soluble, edible, biodegradable, and nontoxic towards humans and the environment, as they degrade into lysine without any side effects (Shukla et al., 2012; Chheda and Vernekar, 2015). Based on its strong antibacterial activity and low toxicity, ε-PL is used as a natural food preservative in Japan, South Korea, United States and China, and it has been currently used in many foods including soft drinks, cheese, egg-based dishes, salad dressings, fish, sauces and potato-based foods (Fadli et al., 2012). Besides its use in the food industry, ε-PL has numerous applications in the pharmaceutical industry as drug carriers, nanoparticles, gene carriers, liposomes, interferon inducers, lipase inhibitors, hydrogels, coating materials, etc. (Bankar and Singhal, 2013).

Nowadays, plenty of efforts and methods have been committed to enhancing ε-PL-producing strains. The most successful one was screening for mutants with S-(2-aminoethyl)-L-cysteine (AEC) plus glycine resistance after nitrosoguanidine treatment and ultraviolet mutagenesis (Hiraki et al., 1998). Moreover, atmospheric and room temperature plasma (ARTP) mutagenesis combined with AEC resistance screening as well as genome shuffling were reported to improve the production of ε-PL (Li et al., 2012; Zong et al., 2012). However, the above methods are quite expensive on account AEC, which is about $180 per gram and rather inefficient. Recently, modern metabolic engineering breeding technology also has been applied in ε-PL production promotion. Xu et al. (2015) established a genetic system to integrate *Vitreoscilla* hemoglobin (VHb) gene into the chromosome of *Streptomyces albulus* PD-1 and Gu et al. (2016) inserted heterologous VHb gene and SAM (S-adenosylmethionine) synthetase gene into the *S. albulus* NK660 chromosome to improve the ε-PL biosynthesis. These methods for improving ε-PL production, ranging from classical random approaches to metabolic engineering, are either costly or labor-intensive. In our lab, Wang et al. (2015, 2017) combined ARTP mutagenesis with streptomycin resistance to obtain a high-yield strain *S. albulus* AS3-14, combined genome shuffling with gentamicin-resistance to achieve the promotion of ε-PL productivity of *S. albulus* W-156 and induced double antibiotic-resistant mutations in *S. albulus*. Despite their efficiency, these methods still present a number of problems, particularly the difficulty in determining the reasons for high yield, due to the complex mutagenesis background.

Genome shuffling (GS) has been extensively used in industry to obtain improved strains, particularly in high GC content microorganisms (Leja et al., 2011). GS combines the advantages of multi-parental crossing facilitated by DNA exchange allowing to access foreign DNA, which facilitates improved phenotypes over traditional random mutagenesis (Stratigopoulos and Cundliffe, 2002). It does not need to know the genetic background of the strain and has the advantage of rearranging the genomes of unknown organisms directly (Zhang et al., 2002). It has become an efficient method of microbial breeding. The first application of GS was the production of tylosin in *Streptomyces fradiae* (Zhang et al., 2002). Subsequently, *Bacillus subtilis*, as riboflavin-producing bacteria, was improved by GS in the laboratory (Chen et al., 2004). GS has become increasingly popular in recent years. Other scholars have successfully selected high-yield enzyme (Liang and Guo, 2007), drug (Gong et al., 2007), food additive (Hida et al., 2007), acid-resistant (Gos and Raszeja, 1993), ethanol-resistant (Macnab, 1996) strains by this method. All these studies used random mutagenesis to generate phenotypic diversity and then recombine with the parental strain by fusing protoplasts.

In this work, we used GS and distinct strains of *S. albulus* obtained by Streptomycin resistance to improve ε-PL production. The mutant strain SG-86 displayed obvious differences in morphology, ε-PL production, and ATP compared to parent strain M-Z18. Illumina sequencing was then used to analyze these two strains and identify novel/unique regions leading to the improved phenotype of recombination strain SG-86. These two strains were sequenced and analyzed and the genome of the recombination strain was about 2.1 M less than that of the parent strain, including a large number of metabolic pathways, secondary metabolic gene clusters, and gene deletions. Using comparative genome analysis, single nucleotide polymorphism (SNPs), insertions and deletions (InDels) analysis (Cingolani et al., 2012), we showed that changes in the genome correspond to InDel and SNP mutations in regions of high recombination probability. Bioinformatics analyses and functional characterizations indicated that the proteins encoded by these mutant genes may be related to ε-PL synthesis. Finally, a mechanism of ε-PL overproduction in *S. albulus* SG-86 was preliminarily constructed by comparative genomics.

## Materials and Methods

### Strains and Media

*Streptomyces albulus* M-Z18 was used as parent strain, whose ε-PL yield was 1.7 g/L in shake-flask fermentation and stored in our lab (Li et al., 2013). *Streptomyces albulus* M-Z18 was isolated from soil as described by Nishikawa and Ogawa (2002) and has been subjected to ultraviolet and nitrosoguanidine mutagenesis as described by Hiraki et al. (1998). Recombination strain *S. albulus* SG-86 was obtained by ribosome engineering and GS. The solid medium (BTN), seed culture medium (M3G), and fermentation medium (YH) were described previously (Liu et al., 2019a). Liquid regeneration medium consisted of 118 g/L sucrose, 25 g/L glucose, 10 g/L MgCl_2_·6H_2_O, 0.4 g/L CaC1_2_·2H_2_O, trace element 2 ml/L, and 10 ml/L TES buffer (pH 7.5). The pH was adjusted to 7.0 with 2 mol/L sterile NaOH after autoclaving. Solid regeneration medium was prepared by adding 20 g agar in 1 L liquid regeneration medium and sterilized at 115°C for 20 min, the pH was adjusted to 7.0 with 2 mol/L NaOH.

### Preparation of the Starting Mutants Through Ribosome Engineering for Genome Shuffling

Spores’ suspension (10^5^ CFU/ml) of *S. albulus* M-Z18 with 120–500 μl was spread on BTN plates containing 0–10 μg/ml streptomycin and incubated at 30°C for 8–10 days. For the primary and secondary screening of mutant strains, multi-well plate fermentation and 250 ml shake-flask fermentation tests were performed as described in our previous study (Liu et al., 2017) and YH was used as fermentation medium. Colonies with abundant spores were inoculated into 24-deep-well microtiter plates containing 2 ml YH medium for 4 days. The mutants with higher ε-PL production were continually inoculated on BTN medium and cultivated for 8–10 days at 30°C until spores mature. Three loops of spores were transferred to 40 ml of M3G medium in a 250 ml flask and subsequently incubated in a rotary shaker (HYL-C, Qiangle Laboratory Equipment Co., Taicang, China) at 200 rpm, 30°C for 24 h. Then, 8.0% (v/v) of seed culture was transferred to fresh YH medium and incubated at 30°C with 200 rpm shaking for 72 h. Five mutants with the highest ε-PL production determined in shake-flask fermentation were chosen as starting strains using 0–45 μg/ml streptomycin to further improve the production of ε-PL (Liu et al., 2019b).

### Protoplast Formation, Fusion, and Regeneration

Ten-milliliter mycelia of strains with high ε-PL production obtained by ribosome engineering were harvested from the logarithmic growth phase, respectively. Then centrifuged (4,500 × *g*) and washed successively with aseptic water and PB buffer. The obtained mycelia were subsequently treated with 5 ml PB buffer and 800 μl lysozyme (50 mg/ml) at 30°C for 2 h, during which the mixed solution is inverted about every 10 min. Protoplasts formation was observed by microscopy and washed twice with PB buffer to remove the lysozyme. Subsequently, 5–6 ml protoplast was inactivated by ultraviolet radiation for 80 min at a distance of 8 W ultraviolet lamp 30 cm in a sterile culture dish with a diameter of 9 cm. The other 5–6 ml protoplasts were inactivated in a 70°C water bath. Protoplasts inactivated by ultraviolet radiation and heat were mixed and centrifuged for 5–10 min at 1,000 rpm. Protoplast fusion was carried out at 38°C–40°C to add 3 ml PEG 6000 for 15 min. The fusions of 180 μl were added in a 5 ml liquid regeneration medium and spread on solid regeneration medium plates. ε-PL productivities of shuffled colonies were evaluated in 24-deep-well microtiter plates and shaking-flask fermentation.

### Morphological Characterization of Strain by Scanning Electron Microscopy

Scanning electron microscopy (SEM) was used to observe the mycelia and spores of *S. albulus* M-Z18 and SG-86 according to Liu et al. (2019a). In brief, cut agar blocks were washed twice in phosphate-buffered saline solution and then immersed in 5% glutaraldehyde. After that, the samples were immobilized with 1% osmium acid (0.1 M PBS, pH 7.2) and then washed with 0.1 M PBS. Then an ethanol gradient dehydration, samples were taken at the critical point dried, and pasted on the sample table of the ion sputtering instrument (SCD 005, BAL-TEC, California, United States) was coated and scanned under the scanning electron microscope (Quanta 200, FEI, Hillsboro, United States).

### Measurement of Intracellular ATP and Proteins

Intracellular ATP was determined as described by Wu et al. (2012) and Wang et al. (2020). And protein synthesis activity *in vivo* according to Myronovskyi et al. (2011) with slight modification. The plasmid PIB139-gusA* was introduced into *Escherichia coli* strain ET12567/pUZ8002 and then transferred into *S. albulus* M-Z18 and SG-86 by intergeneric conjugation to generate M-Z18-GUS and SG-86-GUS, respectively. Mycelia were harvested by centrifugation, washed once with distilled water, and resuspended in lysis buffer [50 mM phosphate buffer (pH 7.0), 5 mM dithiothreitol (DTT), 0.1% Triton X-100, 1 mg/ml lysozyme]. Lysis was performed at 37°C for 15 min. Lysates were centrifuged at 4,000 *g* for 10 min. Then, 0.5 ml of lysate was mixed with 0.5 ml of dilution buffer [50 mM phosphate buffer (pH 7.0), 5 mM DTT, 0.1% Triton X-100] supplemented with 5 ml 0.2 M p-nitrophenyl-b-d-glucuronide. The optical density at 415 nm was measured after 20 min of incubation at 37°C. As a reference, a 1:1 mixture of lysate and dilution buffer was used (Liu et al., 2019a).

### Sequencing and *de novo* Assembly

Total DNA of *S. albulus* M-Z18 and *S. albulus* SG-86 at 30 h during fermentation were extracted and purified by DNA extraction kit (DP302, TIANGEN, Beijing, China) following the manufacturer’s instructions. The quality of the DNA was assessed by gel electrophoresis. And the genome of M-Z18 and SG-86 were sequenced using a PacBio RS II platform at Beijing Genomics Institute (BGI, Shenzhen, China). Four SMRT cells Zero-Mode Waveguide arrays of sequencing were used by the PacBio platform to generate the subreads set. PacBio subreads (length < 1 kb) were removed. The program Pbdagcon was used for self-correction. Draft genomic unitigs, which are uncontested groups of fragments, were assembled using the Celera Assembler against a high-quality corrected circular consensus sequence subreads set. To improve the accuracy of the genome sequences, GATK and SOAP tool packages (SOAP2, SOAPsnp, and SOAPindel) were used to make single-base corrections. To trace the presence of any plasmid, the filtered Illumina reads were mapped using SOAP to the bacterial plasmid database.

### Gene Prediction and Annotation

Putative protein-coding sequences (CDSs) were predicted by Glimmer 3.02 software (Delcher et al., 1999; Olano et al., 2008). tRNA, rRNA, and sRNAs recognition made use of tRNA scan-SE (Lowe and Eddy, 1997), RNAmmer, and the Rfam database. The tandem repeats annotation was obtained using the Tandem Repeat Finder, and the minisatellite DNA and microsatellite DNA were selected based on the number and length of repeat units. CDS annotation was based on the BLASTP program with NR, COG, and KEGG databases. The tRNA genes were directly predicted by tRNAscan-SE (Lowe and Eddy, 1997). The single nucleotide variations (SNPs), InDels, and secondary metabolic gene clusters were identified by Mummer, Mauve, antiSMASH 6.0.1, and BLASTP programs.

### Analytical Methods

The withdrawn fermentation broth was centrifuged at 4,500 *g* for 10 min, the supernatant was used to determine the ε-PL concentration using the methyl orange precipitation method according to Itzhaki (1972). In brief, an equal volume of sample with 0.06–0.12 g/L ε-PL diluted with 0.07 mM phosphate buffer (pH 6.90) and 0.7 mM methyl orange solution were mixed together, which were reacted at 30°C with shaking for 30 min. The ε-PL concentration can be estimated from the absorbance at 465 nm of the methyl orange remaining in the supernatant solution through standard curve calculation.

### Statistical Analysis

All described experiments were performed in independent biological triplicates unless stated otherwise. Presented data in the graphs are the averages of the replicates ± the SD.

## Results and Discussion

### Screening for Improved ε-PL Producing Strains

**Figure 1:**
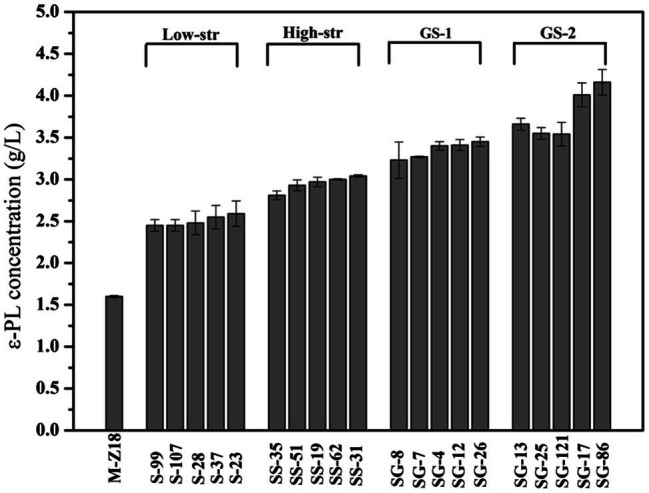
The ε-poly-L-lysine (ε-PL) production of mutants obtained by streptomycin resistance and genome shuffling (low-str, low streptomycin resistance; high-str, high streptomycin resistance; GS-1, first round of genome shuffling; and GS-2: second round of genome shuffling).

Genome shuffling amplifies the genetic diversity within a selected mutant population through extensive homologous recombination. Thus, the starting strains for genome shuffling require a population of improved mutants with diverse genetic sequences (Zhang et al., 2002; Hida et al., 2007). In this work, streptomycin resistance was used for mutation treatment to screen high ε-PL producing strains. As shown in Figure 1, after low streptomycin resistance (0–10 μg/ml streptomycin) five high-yield mutants (S-99, S-107, S-28, S-37, and S-23) were isolated for the secondary screening. The ε-PL productivity of these five mutants in shake flask was 2.45, 2.45, 2.48, 2.55, and 2.59 g/L, respectively. In order to fully release the potential of streptomycin in increasing ε-PL production of *S. albulus*, spores of the high-yield mutants S-99, S-107, S-28, S-37, and S-23 were mixed and spread onto plates containing high streptomycin resistance (0–45 μg/ml streptomycin). Similarly, five high-yield mutants (SS-35, SS-51, SS-19, SS-62, and SS-31) were identified in shake-flask fermentation. The ε-PL productivity of these five mutants in shake flask was 2.81, 2.93, 2.97, 3.00, and 3.04 g/L, respectively. Further genome shuffling SS-35, SS-51, SS-19, SS-62, and SS-31 were used as starting strains. Two successive rounds of genome shuffling were carried out with the mutants. From the first to second rounds of genome shuffling, 324 and 116 colonies appeared on the agar plates, respectively. A 4.16 g/L ε-PL recombinant, SG-86, was obtained after the second round of protoplast fusion, which was 144.7% higher than that of the parent strain M-Z18.

### Phenotypic Difference Between the Wild-Type of *Streptomyces albulus* M-Z18 and *Streptomyces albulus* SG-86

**Figure 2:**
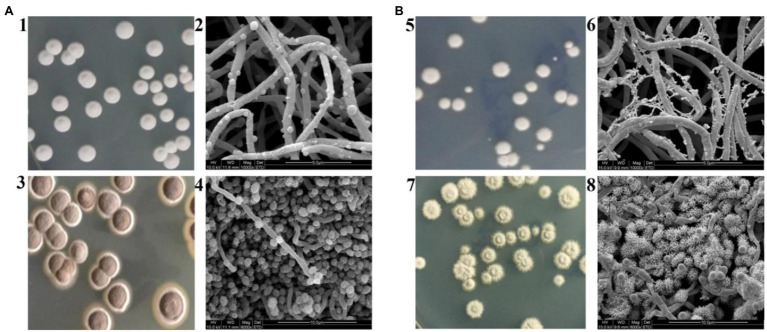
Cultivation characteristics of M-Z18 and SG-86 in solid culture. **(A)** M-Z18 and **(B)** SG-86. 1, 3, 5, 7: colony characterized and spore colour by a camera and 2, 4, 6, 8: hypha and spore characterized by scanning electron microscope (SEM).

Even though *S. albulus* M-Z18 and *S. albulus* SG-86 share a close relationship, they show obvious differences in morphology except for ε-PL production. Figure 2 shows the macroscopic and microscopic photographs of M-Z18 and SG-86. M-Z18 colonies were smooth and its spores were brown (Figures 2A1,3), while SG-86 colonies had a volcanic rock shape with a central depression and were radiation and their spores were greyish green (Figures 2B5,7). SEM analysis showed the hyphae of SG-86 appeared full and abundant as the parent strain M-Z18, but the vesicles became small and the existence of dried mycelium was observed in SG-86 (Figures 2A2,B6). Further, SG-86’s spores were bigger and had more spines than the parent strain M-Z18. What’s interesting was that the vesicles became as large as the mycelium of M-Z18 (Figures 2A4,B8). It is of great value to recognize changes in the cultivation characteristics of high-yielding strain for industrial application (Ren et al., 2015; Wang et al., 2016).

### ATP Levels and Protein Synthesis of *Streptomyces albulus* M-Z18 and *Streptomyces albulus* SG-86

**Figure 3:**
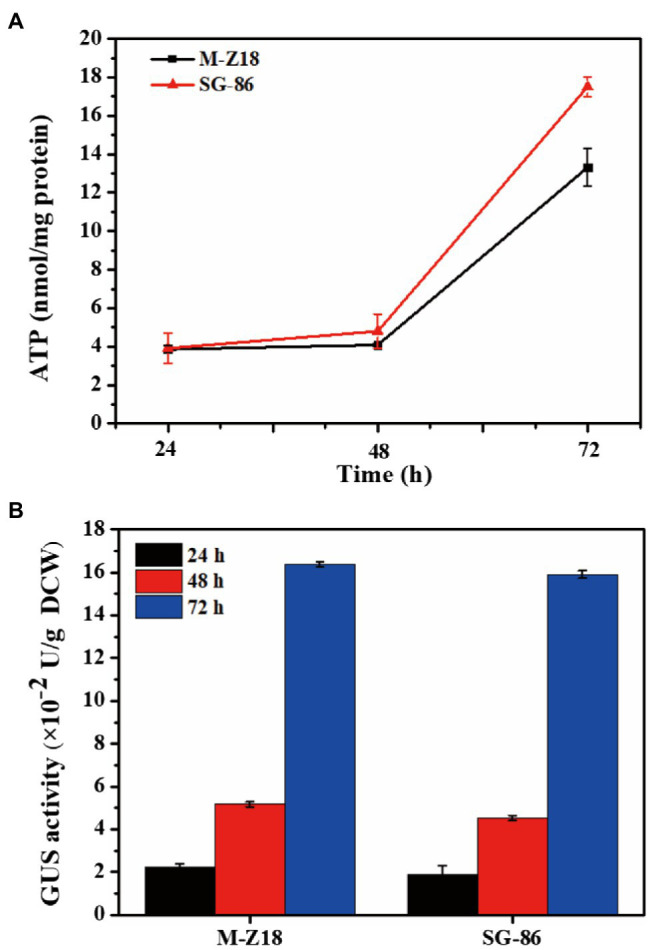
Time-dependent changes of ATP involved in ε-PL biosynthesis in *S. albulus* M-Z18 and mutants SG-86 during shake flask fermentation **(A)**. Detection of b-glucuronidase activity. Glucuronidase activity was measured in cell lysates of parental strain *S. albulus* M-Z18 and mutant SG-86 from fermentation broth at 24, 48, and 72 h, respectively **(B)**.

The process of ε-PL biosynthesis is influenced by energy provision (Yamanaka et al., 2010; Zeng et al., 2014). To understand the intracellular energy, we determined intracellular ATP levels at 24, 48, and 72 h. Figure 3A displayed the results, which represented ATP production from the TCA cycle. SG-86 presented high ATP levels at 48 and 72 h. Especially at 72 h, the intracellular ATP level of SG-86 was 1.31-fold higher than parent strain *S. albulus* M-Z18. We also determined the relationship between protein synthesis and ε-PL production in the parental strain and mutant to use a reporter gene assay based on the glucuronidase gene (Myronovskyi et al., 2011). The glucuronidase activity was measured at 24, 48, and 72 h after inoculation. As shown in Figure 3B, glucuronidase activity displayed no significant difference between mutant SG-86 and parental strain at 48 and 72 h. It indicated that the high-level ε-PL production was not caused by accelerated protein synthesis.

### Illumina Sequencing, Gene Prediction, and Function Annotation

To understand the genetic basis underlying phenotypic differences, especially the apparent differences in the yield of ε-PL and the morphology, between the wild-type *S. albulus* M-Z18 and the high-yield strain *S. albulus* SG-86, the whole genome of *S. albulus* M-Z18 and *S. albulus* SG-86 were sequenced. The complete genome sequence of M-Z18 and SG-86 were determined by *de novo* assemble.

The genomic sequences of the two strains were all composed of two contigs and one plasmid. Similar to the other sequenced *S. albulus*, M-Z18 has a linear chromosome of 9,556,033 bp with an average 72.22% G + C content. A total of 8,897 protein-coding sequences (CDS), 68 tRNA, 52 sRNA, 7 16S-23S-5SrRNA operons, 1707 tandem repeats, 1,159 Minisatellite DNA Numbers, and 232 Microsatellite DNA Numbers were determined by Glimmer 3.02. The chromosome of SG-86 was 7,471,099 bp with an average 72.42% GC content. There were 6,792 CDS, 53 tRNA, 4 16S-23S-5SrRNA operons with a total length of 6,466,035 bp, 1,481 tandem repeats, 1,022 Minisatellite DNA Numbers, and 189 Microsatellite DNA Numbers (Table 1). Moreover, gene prediction and functional annotation by 13 databases including COG, GO, KEGG, NR, and so on. And the proportion of each database is different (Supplementary Table 1).

**Table 1:** General features of genomes of *Streptomyces albulus* M-Z18 and *Streptomyces albulus* SG-86.

	*S. albulus* M-Z18	*S. albulus* SG-86
Genome size(bp)	9,556,033	7,471,099
GC content (%)	72.22	72.42
Gene number	8,897	6,792
Gene total length (bp) gene length/genome (%)	8,282,598 86.67	6,466,035 86.55
tRNA	68	66
rRNA	7 × (16S-23S-5S)	7 × (16S-23S-5S)
sRNA	52	33
Tandem repeat number	1,707	1,481
Minisatellite DNA number	1,159	1,022
Microsatellite DNA number	232	189
Prophage number	2	2
Plasmid	1	1
Plasmid size (bp)	36,936	36,955
Plasmid GC content in %	68.91	68.92

### Genome Variation Analysis of *Streptomyces albulus* M-Z18 and *Streptomyces albulus* SG-86

**Figure 4:**
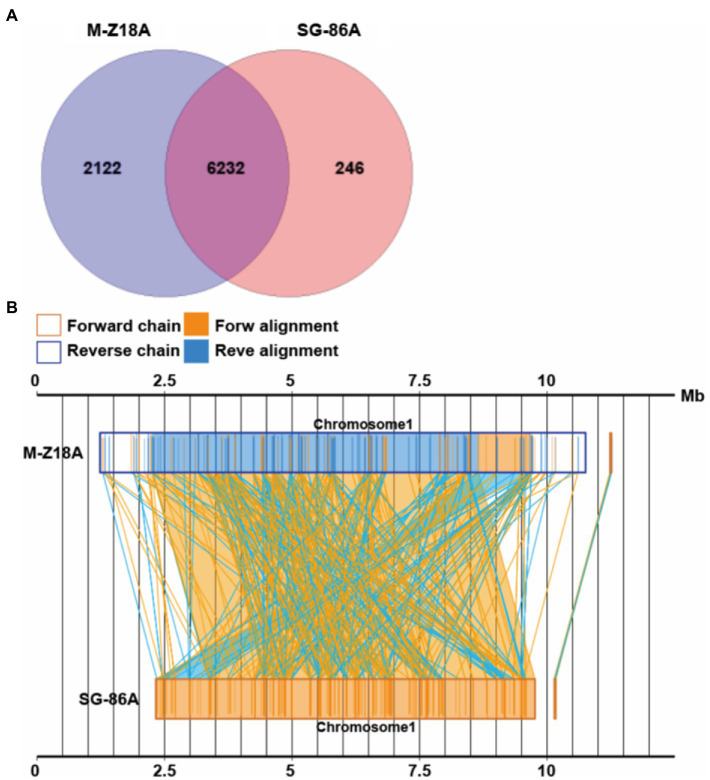
Venn diagram showing the number of core genes and pan genes for two strains **(A)**. Syntenic dot plot of two strains **(B)**.

To elucidate the relationship between phenotype and genotype, we performed a systematic genomic comparison between M-Z18 and SG-86. A gene–gene comparison suggests that M-Z18 has 2,122 unique genes and SG-86 has 246 unique genes (*E*-value < 0.0001; Figure 4A). As a major difference, M-Z18 and SG-86 were not highly conserved gene content and gene order, and there is rearrangement and inversion (Figure 4B). Compared with M-Z18, the chromosome size of SG-86 is 2.1 M smaller and the plasmid size of SG-86 is just 19 bp less than M-Z18 (Table 1). The results showed that the reduced 2.1 M of the SG-86 genome was almost on the chromosome, including metabolic pathways, secondary metabolic gene clusters, and genes. In addition to this large deletion, a total of 142 insertions, 16 deletions, and 33 single nucleotide mutations (SNP) were confirmed. These large deletions, InDels, and SNP will be described in detail below.

### Intragenic InDels Analysis of *Streptomyces albulus* SG-86

Compared with M-Z18, 16 deletion sites and 142 insertion sites were found in SG-86, including 74 intragenic InDels (Supplementary Table 2). Besides the putative and unknown proteins, the proteins encoded by these genes mainly include membrane proteins, ABC transporters, cytochrome P450, and DNA polymerase. In addition, there are two histidine kinases, two types I PKS and one NRPS. The functions of membrane proteins are multifaceted. Membrane proteins play a very important role in many life activities of organisms, such as cell proliferation and differentiation, energy conversion, signal transduction, and material transport. It is estimated that about 60% of the drug targets are membrane proteins (Santos et al., 2016). Besides membrane proteins, some mutations related to transporters were also found, such as 2961361G, 2961427C, and 5685423C. The biological transport system helps cells obtain the required nutrients, excrete harmful products, and the combined function of transporters enables cells to maintain sustainable growth and development. In particular, ABC transporters can increase the availability of nutrients or reduce the number of toxic elements in cells. Transporters are an important factor in anti-phenotypic characteristics including prokaryotes (Padan, 2009). Moreover, transporters can improve acid tolerance. For example, arginine/ornithine reverse transporter is used to introduce decarboxylase reverse transporter of glutamate in arginine deaminase pathway γ-aminobutyrate. Therefore, it is speculated that transporter mutations play a key role in improving pH tolerance (Guan et al., 2013).

Histidine kinase is an important part of the two-component system. The two-component system is a very important regulatory factor, which is responsible for regulating physiological metabolic processes such as cell growth, differentiation, and metabolism, especially closely related to morphological differentiation and secondary metabolite synthesis in Streptomyces (Mendes et al., 2007). Interestingly, four mutations encoding cytochrome P450 were also found. Research shows that P450 is a key participant in the biosynthesis pathway of natural products. Natural products are the most chemically and structurally diverse small molecules known, requiring a large number of P450 (Girvan and Munro, 2016). *Streptomyce avermitilis* and *Streptomyces coelicolor* sequencing found 33 and 18 P450, respectively, accounting for 0.2% and 0.4% of all coding sequences. P450 is usually related to secondary metabolism or biotransformation. The number and diversity of P450 in Streptomyces may affect the biosynthetic potential of natural products (Rudolf et al., 2017). In addition, two genes encoding type I PKS and one NRPS were found to be mutated. The functions of these proteins are unknown, but the results show that they do not affect the yield of ε-PL. It may be beneficial to the synthesis of ε-PL in some way.

### Intergenic InDels Analysis of *Streptomyces albulus* SG-86

As shown in Supplementary Table 3, there are 84 intergenic InDels in SG-86, of which 44 mutations are unknown and hypothetical proteins, accounting for 59.5%. The remaining gene mutations are mainly related to DNA polymerase, transporter, transcription regulatory protein, and methyltransferase. In addition, there are three mutations related to anti macrophage protein, anti-bleomycin protein, and cyclase, respectively. DNA polymerase is associated with genome rearrangement, homologous recombination, gene transformation, or DNA repair (Roth et al., 1996; McVean et al., 2002; Luna-Flores et al., 2017). Mutation-related transporters are mainly ABC transporters, such as ABC transporter ATP binding protein, sugar ABC transporter, and iron carrier ABC transporter. ABC transporters are a class of primary active transporters, including endotransporters and efflux proteins. Endotransporters are mainly responsible for the intake of nutrients and efflux proteins are responsible for transporting substances from intracellular to extracellular. They are mainly related to the efflux of toxic substances such as antibiotics. In Streptomyces, secondary metabolites are secreted extracellularly mainly through ABC transporters (Méndez and Salas, 2001). In addition, ABC transporter is also related to mycelial differentiation and antibiotic synthesis in Streptomyces, which is the link between carbon source utilization, mycelial differentiation, and secondary metabolite synthesis. Hillerich and Westpheling (2006) found that agl3EFG can respond to the signal of glucoside, thus affecting the morphological differentiation and antibiotic synthesis of *S. coelicolor*. Therefore, it is indicated that the mutations related to the transporter may improve ε-PL production of the SG-86 strain.

Mutations are also found in transcription factors. These transcription factors interact with genetics to regulate gene expression. The interaction between protein and DNA may also contribute to new regulatory systems. For example, the transcriptional regulatory TrmB has an insertion mutation. TrmB has been widely studied in archaeococcus, which is closely related to sugar transport and metabolism (Lee et al., 2008). In addition, three mutations are related to transferase, which can catalyze various chemical functional groups except for hydrogen (transfer from one substrate to another), and also play a role in peptide chain extension in protein synthesis. Pisciotta et al. (2018) also found that SCO1731 methyltransferase can regulate the production of actinomycin and morphological differentiation in *Streptomyces coeruleus* A3(2). Whether it plays the same role in SG-86 needs to be further verified.

### SNP Mutation Analysis of *Streptomyces albulus* SG-86

In addition to these InDel mutations, 33 SNP were identified between the two genomes, including 25 intragenic SNPs and 5 intergenic SNPs (Table 2). The intragenic SNPs included 11 synonymous mutations, 1 nonsense mutation, 15 nonsynonymous mutations. In addition to the putative proteins, these 15 nonsynonymous mutations consisted of oxidoreductase, keto acid-reducing isomerase, peptidase, cytochrome P450, transcriptional regulators, transporters, and membrane proteins. Except for anti-σ factor and AfsR family transcription regulatory proteins, the roles of most other proteins have been described before. σ factor is a non-specific protein that acts as a cofactor of all RNA polymerases and it is an inherent component of DNA-dependent RNA polymerase. It recognizes the common sequence of promoters and binds to holoenzyme. σ factor itself cannot bind to DNA, but the interaction with the core enzyme will activate its DNA binding region. Anti-σ factors are regulated by direct interactions between proteins (anti-σ factors and σ factor; Wang et al., 2020). AfsR family regulatory proteins are global regulatory proteins, and AfsK-AfsR can regulate secondary metabolites globally (Horinouchi, 2003). In addition, Sohng (2009) showed that heterologous expression of AfsR homologous genes in other Streptomyces will increase the yield of corresponding antibiotics.

**Table 2:** Single nucleotide mutation of recombinant strain.

Gene ID	Base change	Code change	Change of amino acids	Mutate type	Gene name
Intergenic	T → C				
Intergenic	G → T				
M-Z18AGL001581	G → A	CGG → CGA	R → R	Synonymous	Amidohydrolase
M-Z18AGL001707	C → G	TCC → TGC	S → C	Nonsynonymous	Hypothetical protein
M-Z18AGL001926	T → C	TAC → CAC	Y → H	Nonsynonymous	Oxidoreductase
M-Z18AGL002480	G → T	GCC → GCA	A → A	Synonymous	Acyl-CoA dehydrogenase
M-Z18AGL002891	T → G	GAG→GCG	E → A	Nonsynonymous	Ketol-acid reductoisomerase
M-Z18AGL002996	G → A	GCG → GCA	A → A	Synonymous	Two-component sensor histidine kinase
M-Z18AGL003566	G → C	GGC → GCC	T → A	Nonsynonymous	Peptidase
Intergenic	G → A				
M-Z18AGL003702	C → T	GAG→AAG	E → K	Nonsynonymous	Cytochrome P450
M-Z18AGL003817	G → A	AAG → AAA	K → K	Synonymous	Hypothetical protein
M-Z18AGL004063	A → T	TCA → ACA	S → T	Nonsynonymous	Anti-sigma factor antagonist
M-Z18AGL004389	C → T	CTC → CTT	L → L	Synonymous	Membrane protein
M-Z18AGL004492	C → T	GAA → AAA	E → K	Nonsynonymous	AfsR family transcriptional regulator
M-Z18AGL004926	G → A	CTG → CTA	L → L	Synonymous	Hypothetical protein K530_49370
M-Z18AGL004926	G → A	GCG → ACG	A → T	Nonsynonymous	Hypothetical protein K530_49370
M-Z18AGL004963	T → A	CAG → CTG	Q → L	Nonsynonymous	Transcriptional regulator
Intergenic	C → T				
M-Z18AGL005160	G → A	CCG → CCA	P → P	Synonymous	Hypothetical protein
M-Z18AGL005326	T → C	GTC → GCC	V → A	Nonsynonymous	Membrane protein
M-Z18AGL005622	C → G	CGG → CCG	R → P	Nonsynonymous	6-Phospho-beta-glucosidase
M-Z18AGL005910	C → T	GTG → GTA	V → V	Synonymous	DNA-binding protein
M-Z18AGL006102	G → A	GCC → ACC	A → T	Nonsynonymous	Transporter
M-Z18AGL006129	T → A	CTC → CAC	L → H	Nonsynonymous	Tryptophan synthase subunit alpha
M-Z18AGL006373	C → T	CGG → CGA	R → R	Synonymous	Protease
M-Z18AGL007131	C → T	GCC → GCT	A → A	Synonymous	Oxidoreductase
M-Z18AGL007294	C → T	CTG → TTG	L → L	Synonymous	Type I polyketide synthase
M-Z18AGL007603	T → G	CTC → CGC	L → R	Nonsynonymous	Cyclase
M-Z18AGL007672	G → A	CAG → TAG	Q → X	Nonsense	Transporter
M-Z18AGL008875	C → T	GGG → AGG	G → R	Nonsynonymous	Transcriptional regulator
Intergenic	T → G				
Intergenic	G → T				

### Metabolic Pathway and Gene Deletion Analysis of *Streptomyces albulus* SG-86

According to KEGG analysis, both M-Z18 and SG-86 have complete EMP (glycolytic pathway), PPP (pentose phosphate pathway), TCA (tricarboxylic acid cycle), and DAP (diaminopimelate pathway) metabolic pathways, and have efficient material and energy metabolism in the fermentation process. However, SG-86 has a large number of gene deletions among these metabolic pathways, including the deletion of metabolic pathways. Supplementary Table 4 shows that SG-86 lacks four metabolic pathways, namely sterol synthesis, phosphatidylinositol signaling system, Salmonella infection, and sterol biosynthesis. These pathways have nothing to do with ε-PL synthesis, which indicated that genome rearrangement removes many metabolic pathways unrelated to ε-PL synthesis in SG-86, reduces the genome of SG-86, and may improve the efficiency of intracellular ε-PL synthesis. In addition, there are gene deletions in alanine and glutamate (racD), glycine, serine and threonine (Gatm), arginine and proline (E3.5.2.10), histidine (DDC), tyrosine (moaA), tryptophan (DDC) metabolic pathways, and valine, leucine, and isoleucine (IVD, scoB) degradation pathways (Supplementary Table 4). The loss of these by-product metabolism and degradation pathway genes reduces the synthesis of by-products, and more metabolic flow flows to the precursor of ε-PL, which may improve the production of ε-PL.

### Secondary Metabolic Gene Clusters Deletion Analysis of *Streptomyces albulus* SG-86

**Figure 5:**
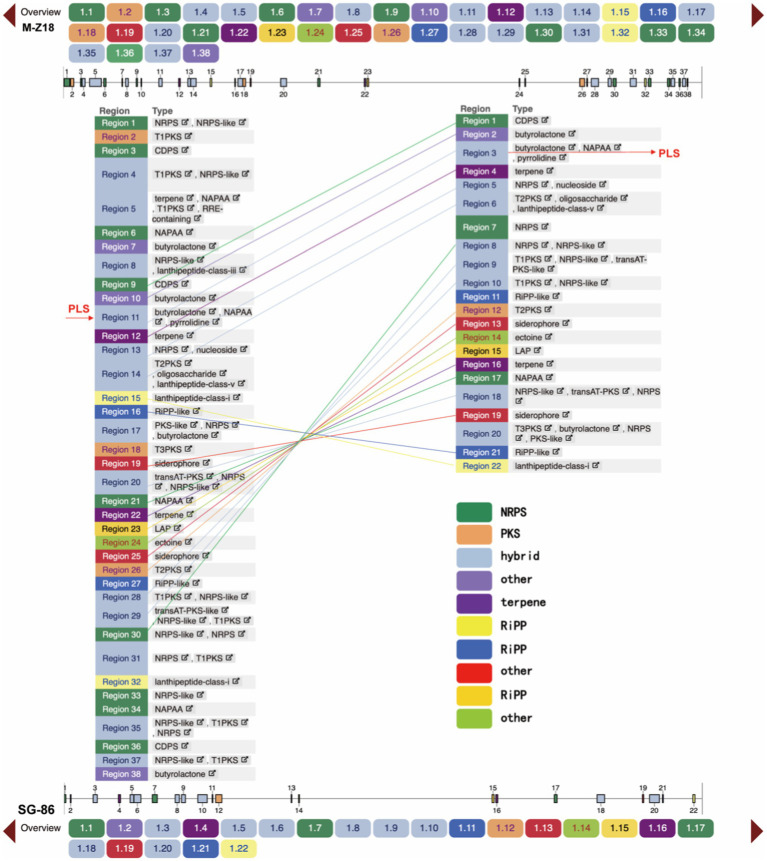
Secondary metabolic gene clusters analysis of M-Z18 and SG-86.

We predicted the secondary metabolic gene clusters of the two strains by antiSMASH 6.0.1 and the results showed that there were 38 secondary metabolic gene clusters in M-Z18, including nine non-ribosomal peptide synthetase (NRPS), three polyketide synthase (PKS; T1PKS, T2PKS, and T3PKS), 13 hybrid gene clusters, three terpenes, four Ripp (Other unspecified ribosomally synthesized and post-translationally modified peptide product cluster) and seven other secondary metabolic gene clusters. However, only 22 secondary metabolic gene clusters were found in SG-86, including three NRPS, one type II PKS, eight hybrid synthetase, two terpenes, four Ripp, and four other secondary metabolic gene clusters. SG-86 had six NRPS, two PKS, five hybrid gene clusters, one terpene, and three other secondary metabolic gene clusters less than M-Z18. After comparison, it was found that these missing gene clusters were at both ends and far away from ε-PL synthase (PLS) in M-Z18 (Figure 5). In Actinomycetes, the synthesis of secondary metabolites is catalyzed by a variety of enzymes, the most important of which are PKS and NRPS, while the missing PKS and NRPS in SG-86 do not affect ε-PL yield. On the contrary, the deletion of secondary metabolic gene clusters makes some other compounds no longer produced, and more energy and carbon metabolism flow may flow to ε-PL production.

## Conclusion

**Figure 6:**
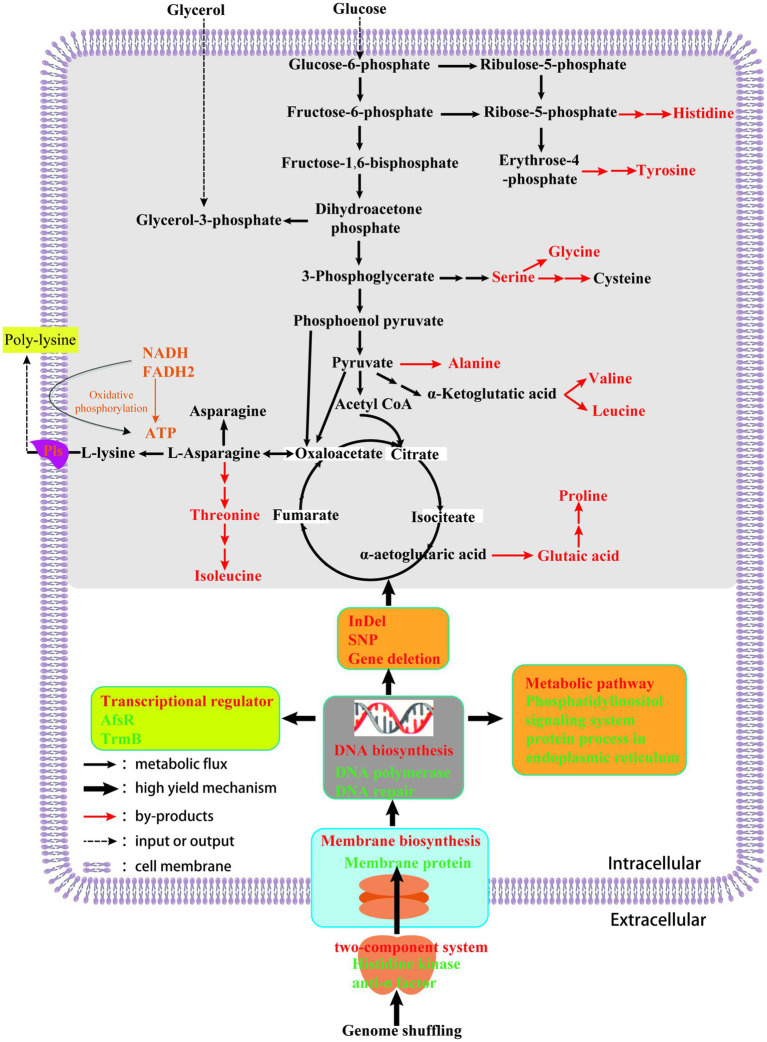
High-yielding mechanism of ε-PL from *S. albulus* using genome shuffling.

Based on the above results, an ε-PL high yield mechanism of *S. albulus* was preliminarily proposed (Figure 6). In this work, we used genome shuffling on strains obtained by ribosome engineering to obtain an ε-PL high yield mutant strain. The mutant strain SG-86 displayed obvious differences in morphology, ε-PL production, and ATP compared to parent strain M-Z18. Except that the two-component system histidine kinase and anti-σ factor mutated after GS. Anti-σ factor blocking the interaction between σ factor and RNA polymerase core enzyme leads to the increase of transcription level of genes related to peptidoglycan synthesis, to guide the correct synthesis of the cell wall. Meanwhile, the selective permeability of the cell membrane was changed. Four genes encoding membrane proteins have InDels. Membrane proteins play a very important role in many life activities of organisms, such as cell proliferation and differentiation, energy conversion, signal transduction, and material transport. Besides, DNA synthesis and repairability changed. The gene encoding DNA polymerase and DNA polymerase subunit is mutated, but DNA replication is normal, which indicates that the enzyme has little relationship with DNA replication and plays an important role in DNA repair. The level of transcriptional regulation changed. For example, TrmB, AfsK, and other genes are mutated. AfsK can globally regulate secondary metabolites, and these transcriptional regulators interact with genetic materials to regulate gene expression, which may also contribute to a new regulatory system. In addition, GS leads to a large number of deletions in genes, secondary metabolic gene clusters, and metabolic pathways. Genome “shrink” not only removes some redundant genes, but also leads to the reduction of the synthesis of metabolic by-products such as alanine, glutamate, glycine, serine, threonine, arginine, and histidine. And we predicted more metabolic may flow to the precursor of ɛ-PL. Then, the production of ε-PL was eventually promoted. More importantly, the analysis of ε-PL high yield mechanism provides theoretical guidance for metabolic engineering transformation to further improve the production of ε-PL.

## Data Availability Statement

The original contributions presented in the study are included in the article/Supplementary Material, further inquiries can be directed to the corresponding author.

## Author Contributions

XC conceived the project and supervised the research. XC and YL designed the experiments, analyzed all data, and wrote the manuscript. YL, KW, and LP performed the experiments. All authors contributed to the article and approved the submitted version.

## Funding

This work was financially supported by the National Key R&D Program of China (2020YFA0907700), the National Natural Science Foundation of China (31901622 and 32100049), and the Natural Science Foundation of Jiangsu Province (BK20191332 and BK20190585).

## Conflict of Interest

The authors declare that the research was conducted in the absence of any commercial or financial relationships that could be construed as a potential conflict of interest.

## Publisher’s Note

All claims expressed in this article are solely those of the authors and do not necessarily represent those of their affiliated organizations, or those of the publisher, the editors and the reviewers. Any product that may be evaluated in this article, or claim that may be made by its manufacturer, is not guaranteed or endorsed by the publisher.
