# Supplementary material for: Improved Production of ε-Poly-L-Lysine in Streptomyces albulus Using Genome Shuffling and Its High-Yield Mechanism Analysis
Source: Front Microbiol. 2022 May 31;13:923526. doi: 10.3389/fmicb.2022.923526 (PMC9195005; doi:10.3389/fmicb.2022.923526)
Supplement: Supplementary file 1 [file Table_1.DOCX]

# Additional file

# Improved production of ε-poly-L-lysine in *Streptomyces albulus* using genome shufﬂing and its high-yield mechanism analysis

**Yongjuan Liu^1,2,3,4^ · Kaifang Wang^2^ · Long Pan^2,5^ · Xusheng Chen^2*^**

^1^ Shandong Provincial Key Laboratory of Synthetic Biology, Qingdao Institute of Bioenergy and Bioprocess Technology, Chinese Academy of Sciences, Qingdao 266101, China.

^2^ The Key Laboratory of Industrial Biotechnology, Ministry of Education, School of Biotechnology, Jiangnan University, Wuxi 214122, China

^3^ Shandong Energy Institute, Qingdao, China.

^4^ Qingdao New Energy Shandong Laboratory, Qingdao, China.

^5^ College of Biological Engineering, Henan University of Technology, Zhengzhou, China.

* Correspondence:

Prof. Xusheng Chen, School of Biotechnology, Jiangnan University, 1800 Lihu Road, Wuxi 214122, Jiangsu, China

E-mails: chenxs@jiangnan.edu.cn

Supplementary Table 1 Notes on the database of *S.albulus* M-Z18 and *S.albulus* SG-86

|  | *S.albulus* M-Z18 | *S.albulus* SG-86 |
| --- | --- | --- |
| Total Gene Number | 8,897 | 6,792 |
| VFDB | 367(4.12%) | 291(4.28%) |
| ARDB | 24(0.26%) | 16(0.23%) |
| TREMBL | 8,218(92.36%) | 6,385 (94%) |
| CAZY | 165(1.85%) | 140 (2.06%) |
| PHI | 131(1.47%) | 114 (1.67%) |
| IPR | 6,691 (75.2%) | 5252 (77 .32%) |
| SWISSPROT | 2,456 (27.6%) | 2,078 (30.59%) |
| COG | 5,822(65.43%) | 4,626 (68.1%) |
| CARD | 122 (1.37%) | 97 (1.42%) |
| GO | 4,804 (53.99%) | 3,774 (55.56%) |
| KEGG | 3,800 (42.71%) | 3,080 (45.34%) |
| NR | 8,302 (93.31%) | 6,427 (94.62%) |
| T3SS  OverAll | 1,868 (20.99%)  8,474 (95.24%) | 1,426 (20.99%)  6,525 (96.06%) |

Supplementary Table 2 Intragenic InDel mutation in SG-86 compared with M-Z18

| InDelType | InDelStart SG-86 | InDelEnd SG-86 | InDelStart M-Z18 | InDelEnd M-Z18 | InDelBase | Genen name |
| --- | --- | --- | --- | --- | --- | --- |
| Insertion | 173088 | 173089 | 6674966 | 6674966 | G | M-Z18AGL006167 |
| Insertion | 233772 | 233773 | 2049839 | 2049839 | G | Dehydrogenase |
| Insertion | 269191 | 269192 | 2014423 | 2014423 | C | Von Willebrand factor A |
| Insertion | 979636 | 979637 | 1304008 | 1304008 | G | Cytochrome P450 |
| Insertion | 979537 | 979538 | 1304106 | 1304106 | G | Cytochrome P450 |
| Insertion | 19243 | 19246 | 5926047 | 5926047 | GGC | Methylmalonate-semialdehyde dehydrogenase |
| Insertion | 401808 | 401809 | 1881816 | 1881816 | C | RNA polymerase ECF-subfamily sigma factor |
| Insertion | 208903 | 208904 | 4537673 | 4537673 | G | Hypothetical protein |
| Deletion | 262327 | 262327 | 2853085 | 2853094 | TGCTCCTCG | Histidine kinase |
| Insertion | 49231 | 49232 | 2691361 | 2691361 | G | ABC transporter ATP-binding protein |
| Insertion | 49298 | 49299 | 2691427 | 2691427 | C | ABC transporter ATP-binding protein |
| Insertion | 286335 | 286336 | 6788208 | 6788208 | C | Hypothetical protein |
| Insertion | 286418 | 286419 | 6788290 | 6788290 | C | Hypothetical protein |
| Insertion | 255050 | 255051 | 3314815 | 3314815 | G | Hypothetical protein |
| Deletion | 26873 | 26873 | 7720944 | 7720952 | ACCACCGC | Monooxygenase |
| Insertion | 882720 | 882721 | 1400919 | 1400919 | C | Membrane protein |
| Insertion | 126397 | 126398 | 5046805 | 5046805 | C | M-Z18AGL004679 |
| Deletion | 178218 | 178218 | 5572475 | 5572480 | CGGGC | M-Z18AGL005170 |
| Insertion | 1716 | 1717 | 7406186 | 7406186 | C | Flavin-dependent reductase |
| Insertion | 60031 | 60032 | 2540113 | 2540113 | G | Peptidase M15 |
| Insertion | 86382 | 86383 | 8129672 | 8129672 | C | Cytochrome P450 |
| Insertion | 33675 | 33676 | 6535556 | 6535556 | C | 6-phosphofructokinase |
| Insertion | 584460 | 584461 | 1699169 | 1699169 | G | Amidohydrolase |
| Insertion | 223160 | 223161 | 6725035 | 6725035 | C | Phosphonate metabolism protein |
| Insertion | 59878 | 59879 | 2540265 | 2540265 | G | M-Z18AGL002416 |
| Insertion | 23846 | 23847 | 1144837 | 1144837 | C | Carbamoyltransferase HypF |
| Insertion | 418171 | 418172 | 6215257 | 6215257 | G | Regulatory protein |
| Insertion | 358221 | 358222 | 1925395 | 1925395 | G | aldo/keto reductase |
| Insertion | 156282 | 156283 | 5953377 | 5953377 | G | Hypothetical protein |
| Insertion | 78969 | 78970 | 4065893 | 4065893 | C | Hypothetical protein P354_23030 |
| Insertion | 78994 | 78995 | 4065917 | 4065917 | C | Hypothetical protein P354_23030 |
| Insertion | 47789 | 47790 | 2998626 | 2998626 | C | Hypothetical protein |
| Insertion | 578398 | 578399 | 1705229 | 1705229 | G | ATPase |
| Insertion | 327126 | 327127 | 3651699 | 3651699 | G | Hypothetical protein DC74_5181 |
| Insertion | 327206 | 327207 | 3651778 | 3651778 | G | Hypothetical protein DC74_5181 |
| Insertion | 23107 | 23108 | 4235784 | 4235784 | C | Pyridine nucleotide-disulfide oxidoreductase |
| Insertion | 184914 | 184915 | 3509490 | 3509490 | G | Transferase |
| Insertion | 171692 | 171693 | 7588756 | 7588756 | C | M-Z18AGL007008 |
| Insertion | 254640 | 254641 | 3314406 | 3314406 | G | Hypothetical protein |
| Insertion | 14386 | 14387 | 2614709 | 2614709 | C | M-Z18AGL002482 |
| Deletion | 19034 | 19034 | 533599 | 533608 | GCGGGCGGT | Beta-ketoacyl synthase |
| Insertion | 254516 | 254517 | 3314283 | 3314283 | G | M-Z18AGL003043 |
| Insertion | 414377 | 414378 | 6211465 | 6211465 | G | POSSIBLE METHYLTRANSFERASE |
| Insertion | 187706 | 187707 | 7057716 | 7057716 | C | Cytochrome P450 |
| Insertion | 277051 | 277052 | 6968378 | 6968378 | G | DNA polymerase III subunit alpha |
| Insertion | 154093 | 154094 | 6655973 | 6655973 | T | Membrane protein |
| Insertion | 1004281 | 1004291 | 1279365 | 1279365 | GCGGCGGCGG | Exonuclease |
| Insertion | 60199 | 60202 | 1117650 | 1117650 | GCT | Histidine kinase |
| Insertion | 124013 | 124014 | 5044422 | 5044422 | G | Hypothetical protein |
| Insertion | 578928 | 578929 | 1704700 | 1704700 | G | Dynein regulation protein LC7 |
| Insertion | 394965 | 394966 | 6192055 | 6192055 | C | M-Z18AGL005709 |
| Insertion | 275162 | 275163 | 6970266 | 6970266 | C | DNA polymerase |
| Deletion | 35147 | 35147 | 2273690 | 2273696 | GCCGCC | Membrane protein |
| Insertion | 120039 | 120040 | 2926379 | 2926379 | G | M-Z18AGL002736 |
| Insertion | 2342 | 2343 | 7922548 | 7922548 | C | Type I polyketide synthase |
| Insertion | 94663 | 94664 | 8121393 | 8121393 | C | Integral membrane protein |
| Insertion | 19167 | 19168 | 1149515 | 1149515 | G | Hypothetical protein |
| Insertion | 145 | 146 | 8217357 | 8217357 | G | Non-ribosomal peptide synthetase |
| Insertion | 111518 | 111519 | 5685423 | 5685423 | C | ABC transporter permease |
| Insertion | 278627 | 278628 | 6075720 | 6075720 | G | Hypothetical protein DC74_3092 |
| Insertion | 109868 | 109870 | 1047856 | 1047856 | CC | M-Z18AGL000925 |
| Deletion | 109879 | 109879 | 1047866 | 1047873 | GCAAACG | M-Z18AGL000925 |
| Insertion | 10732 | 10733 | 7935717 | 7935717 | C | Type I polyketide synthase |
| Insertion | 182707 | 182708 | 3507285 | 3507285 | C | Reductase |
| Insertion | 182817 | 182818 | 3507394 | 3507394 | C | Reductase |
| Insertion | 146163 | 146164 | 3912957 | 3912957 | C | Hypothetical protein |
| Deletion | 128951 | 128951 | 2661374 | 2661377 | GCC | Methylmalonate-semialdehyde dehydrogenase |
| Insertion | 983041 | 983042 | 1300604 | 1300604 | G | Acetyltransferase |
| Insertion* | 7100 | 7101 | 32055 | 32055 | G | DNA polymerase III subunit epsilon |
| Insertion* | 3079 | 3080 | 36059 | 36059 | G | M-Z18AGL008897 |
| Insertion* | 3034 | 3035 | 36103 | 36103 | G | M-Z18AGL008897 |
| Insertion* | 3022 | 3023 | 36114 | 36114 | G | M-Z18AGL008897 |
| Insertion* | 3010 | 3011 | 36125 | 36125 | C | M-Z18AGL008897 |
| Insertion* | 7100 | 7101 | 32055 | 32055 | G | DNA polymerase III subunit epsilon |

*represent insertion and deletion mutations on plasmid and the others all on chromos

Supplementary Table 3 Intergenic InDel mutation in SG-86 compared with M-Z18

| InDelType | InDelStart SG-86 | InDelEnd SG-86 | InDelStart M-Z18 | InDelEnd M-Z18 | InDelBase | Gene name |
| --- | --- | --- | --- | --- | --- | --- |
| Deletion | 109499 | 109499 | 1047447 | 1047452 | GCGTT | Cupin |
| Deletion | 109524 | 109524 | 1047477 | 1047478 | C | Cupin |
| Insertion | 109611 | 109612 | 1047564 | 1047564 | A | M-Z18AGL000925 |
| Insertion | 966403 | 966404 | 1317238 | 1317238 | G | Patatin |
| Insertion | 785643 | 785644 | 1497994 | 1497994 | C | Cyclase |
| Insertion | 783756 | 783757 | 1499879 | 1499879 | C | Demethylmenaquinone methyltransferase |
| Insertion | 783680 | 783681 | 1499954 | 1499954 | C | Demethylmenaquinone methyltransferase |
| Insertion | 358713 | 358714 | 1924904 | 1924904 | G | Aldo/keto reductase |
| Insertion | 193266 | 193267 | 2090343 | 2090343 | C | Phosphoesterase |
| Insertion | 27743 | 27744 | 2356200 | 2356200 | C | Glyoxalase |
| Insertion | 23891 | 23892 | 2360051 | 2360051 | C | Allantoicase |
| Insertion | 16384 | 16385 | 2367557 | 2367557 | G | Hypothetical protein |
| Insertion | 16235 | 16236 | 2367705 | 2367705 | G | 4-diphosphocytidyl-2C-methyl-D-erythritol synthase |
| Insertion | 3317 | 3318 | 2388936 | 2388936 | C | Hypothetical protein |
| Insertion | 3371 | 3372 | 2388989 | 2388989 | C | Hypothetical protein |
| Insertion | 13048 | 13049 | 2398665 | 2398665 | C | Hypothetical protein |
| Insertion | 13084 | 13085 | 2398700 | 2398700 | C | Hypothetical protein |
| Insertion | 13303 | 13304 | 2398857 | 2398857 | C | Hypothetical protein |
| Insertion | 17804 | 17805 | 2618126 | 2618126 | C | 3-oxoacyl-ACP synthase |
| Insertion | 119890 | 119891 | 2926527 | 2926527 | C | M-Z18AGL002736 |
| Insertion | 93708 | 93709 | 2952708 | 2952708 | C | ABC transporter ATP-binding protein |
| Insertion | 46290 | 46291 | 3000124 | 3000124 | G | Methyltransferase type 11 |
| Insertion | 8900 | 8901 | 3037513 | 3037513 | C | D-alanine--D-alanine ligase A |
| Insertion | 256658 | 256659 | 3316422 | 3316422 | G | Hypothetical protein |
| Insertion | 256745 | 256746 | 3316508 | 3316508 | G | Hypothetical protein |
| Insertion | 326950 | 326951 | 3651524 | 3651524 | G | Hypothetical protein DC74_5181 |
| Insertion | 498036 | 498037 | 3822604 | 3822604 | G | Hypothetical protein |
| Insertion | 147466 | 147468 | 3914472 | 3914472 | GA | Hypothetical protein |
| Insertion | 33316 | 33317 | 4179324 | 4179324 | C | Metallothiol transferase FosB |
| Insertion | 229507 | 229508 | 4558275 | 4558275 | G | Transcriptional regulator, TrmB |
| Insertion | 339804 | 339805 | 5398401 | 5398401 | C | M-Z18AGL005014 |
| Insertion | 339941 | 339942 | 5398537 | 5398537 | C | M-Z18AGL005014 |
| Insertion | 153309 | 153310 | 5643635 | 5643635 | G | Phosphoribosyltransferase |
| Insertion | 113208 | 113209 | 5683734 | 5683734 | C | Sugar ABC transporter permease |
| Insertion | 109586 | 109587 | 5687354 | 5687354 | C | Sugar ABC transporter substrate-binding protein |
| Insertion | 278297 | 278298 | 6075391 | 6075391 | G | Hypothetical protein |
| Insertion | 289253 | 289254 | 6086345 | 6086345 | G | Hypothetical protein |
| Insertion | 403171 | 403172 | 6200260 | 6200260 | G | Rod shape-determining protein |
| Insertion | 415705 | 415706 | 6212792 | 6212792 | G | Possible methyltransferase |
| Insertion | 418797 | 418798 | 6215882 | 6215882 | C | Hypothetical protein ADK38_36600 |
| Insertion | 418948 | 418949 | 6216032 | 6216032 | C | Glyoxalase/bleomycin resistance protein/dioxygenase |
| Insertion | 492814 | 492815 | 6289897 | 6289897 | C | Hypothetical protein SAZ_15520 |
| Deletion | 589904 | 589904 | 6354220 | 6354222 | TC | Hypothetical protein |
| Deletion | 588392 | 588392 | 6355521 | 6355522 | G | Hypothetical protein |
| Deletion | 78181 | 78181 | 6398622 | 6398624 | CT | DNA-binding protein |
| Insertion | 87235 | 87236 | 6409591 | 6409591 | A | M-Z18AGL005927 |
| Insertion | 87300 | 87301 | 6409655 | 6409655 | G | M-Z18AGL005927 |
| Insertion | 173007 | 173008 | 6674886 | 6674886 | G | M-Z18AGL006167 |
| Insertion | 288414 | 288415 | 6790285 | 6790285 | G | Hypothetical protein |
| Insertion | 265471 | 265472 | 6979956 | 6979956 | G | Type 11 methyltransferase |
| Insertion | 216312 | 216313 | 7029113 | 7029113 | C | Laminin |
| Insertion | 216165 | 216166 | 7029259 | 7029259 | C | Laminin |
| Insertion | 140861 | 140862 | 7104559 | 7104559 | G | Peptidase M50 |
| Insertion | 140787 | 140788 | 7104632 | 7104632 | C | Transposase |
| Insertion | 93091 | 93092 | 7152327 | 7152327 | A | Hypothetical protein DC74_2077 |
| Insertion | 78483 | 78484 | 7166932 | 7166932 | G | Hypothetical protein |
| Insertion | 118 | 128 | 7426539 | 7426539 | GCGCTTCCCA | Regulatory protein |
| Insertion | 34 | 35 | 7426615 | 7426615 | G | Regulatory protein |
| Insertion | 102009 | 102010 | 8114048 | 8114048 | C | Iron-siderophore ABC transporter |
| Insertion | 93715 | 93716 | 8122340 | 8122340 | G | Hypothetical protein K530_20145 |
| Insertion | 52629 | 52630 | 8163424 | 8163424 | G | M-Z18AGL007488 |
| Insertion | 52289 | 52290 | 8163763 | 8163763 | G | Thiamine biosynthesis lipoprotein |
| Deletion | 838499 | 838499 | 8572731 | 8572737 | CTGCAG | Natural resistance-associated macrophage protein |
| Insertion | 838752 | 838755 | 8572989 | 8572989 | GTG | Natural resistance-associated macrophage protein |
| Insertion* | 5798 | 5799 | 33356 | 33356 | G | DNA polymerase III subunit epsilon |
| Insertion* | 5762 | 5763 | 33391 | 33391 | G | DNA polymerase III subunit epsilon |
| Insertion* | 5733 | 5734 | 33419 | 33419 | G | DNA polymerase III subunit epsilon |
| Insertion* | 5701 | 5703 | 33449 | 33449 | GC | DNA polymerase III subunit epsilon |
| Insertion* | 5618 | 5619 | 33531 | 33531 | G | DNA polymerase III subunit epsilon |
| Insertion* | 5467 | 5468 | 33681 | 33681 | G | Hypothetical protein |
| Insertion* | 5386 | 5387 | 33761 | 33761 | G | Hypothetical protein |
| Insertion* | 5240 | 5241 | 33906 | 33906 | G | Hypothetical protein |
| Insertion* | 5228 | 5229 | 33917 | 33917 | G | Hypothetical protein |
| Insertion* | 4953 | 4954 | 34191 | 34191 | G | Hypothetical protein |
| Insertion* | 4894 | 4895 | 34249 | 34249 | C | Hypothetical protein |
| Deletion* | 3910 | 3910 | 35232 | 35233 | G | M-Z18AGL008897 |
| Insertion* | 3780 | 3781 | 35363 | 35363 | G | M-Z18AGL008897 |
| Insertion* | 3762 | 3763 | 35380 | 35380 | G | M-Z18AGL008897 |
| Insertion* | 3492 | 3493 | 35649 | 35649 | A | M-Z18AGL008897 |
| Insertion* | 3425 | 3427 | 35714 | 35714 | GC | M-Z18AGL008897 |
| Deletion* | 2955 | 2955 | 36179 | 36180 | A | M-Z18AGL008897 |
| Deletion* | 2890 | 2890 | 36245 | 36246 | G | M-Z18AGL008897 |
| Insertion* | 2838 | 2839 | 36298 | 36298 | C | M-Z18AGL008897 |
| Insertion* | 2813 | 2814 | 36322 | 36322 | T | M-Z18AGL008897 |

* represent insertion and deletion mutations on plasmid and the others all on chromosome

Supplementary Table 4 Metabolic pathways and genes deleted in SG-86 strain

| Gene ID | Gene Name | Protein | Metabolic Pathway |
| --- | --- | --- | --- |
| M-Z18AGL000362 | dgoD | galactonate dehydratase | Galactose metabolism |
| M-Z18AGL000795 | fabZ | 3-hydroxyacyl-[acyl-carrier-protein] dehydratase | Fatty acid metabolism |
| M-Z18AGL008149 | ACOX | acyl-CoA oxidase | Fatty acid degradation |
| M-Z18AGL008294 | scoA | 3-oxoacid CoA-transferase subunit A | Synthesis and degradation of ketone bodies |
| - | - | - | sterod biosynthesis |
| M-Z18AGL007917 | E3.1.2.23 | 4-hydroxybenzoyl-CoA thioesterase | Ubiquinone and other terpenoid-quinone biosynthesis |
| M-Z18AGL006218 | petC | cytochrome b6-f complex iron-sulfur subunit | Photosynthesis |
| M-Z18AGL000800 | pyrDI | dihydroorotate dehydrogenase (NAD+) catalytic subunit | Pyrimidine metabolism |
| M-Z18AGL008143 | rutB | ureidoacrylate peracid hydrolase |  |
| M-Z18AGL007833 | racD | aspartate racemase | Alanine, aspartate and glutamate metabolism |
| M-Z18AGL000577 | GATM | glycine amidinotransferase | Glycine, serine and threonine metabolism |
| M-Z18AGL008209 | nocF | 4-hydroxymandelate synthase | Monobactam biosynthesis |
| M-Z18AGL008207 | nocN | 4-hydroxymandelate oxidase |  |
| M-Z18AGL008205 | nocG | (S)-3,5-dihydroxyphenylglycine transaminase |  |
| M-Z18AGL000951 | ivd | isovaleryl-CoA dehydrogenase IVD | Valine, leucine and isoleucine degradation |
| M-Z18AGL008295 | scoB | 3-oxoacid CoA-transferase subunit B |  |
| M-Z18AGL000899 | atuD | citronellyl-CoA dehydrogenase | Geraniol degradation |
| M-Z18AGL000801 | E3.5.2.10 | creatinine amidohydrolase | Arginine and proline metabolism |
| M-Z18AGL000577 | GATM | glycine amidinotransferase |  |
| M-Z18AGL000441 | E5.1.1.4 | proline racemase |  |
| M-Z18AGL008643 | DDC | aromatic-L-amino-acid decarboxylase | Histidine metabolism |
| M-Z18AGL005181 | moaA | GTP 3',8-cyclase | Tyrosine metabolism |
| M-Z18AGL008643 | DDC | aromatic-L-amino-acid decarboxylase |  |
| M-Z18AGL008142 | E1.1.1.90 | aryl-alcohol dehydrogenase |  |
| M-Z18AGL008643 | DDC | aromatic-L-amino-acid decarboxylase | Phenylalanine metabolism |
| M-Z18AGL008353 | katG | catalase-peroxidase |  |
| M-Z18AGL000938 | mhpD | 2-keto-4-pentenoate hydratase |  |
| M-Z18AGL000936 | mhpE | 4-hydroxy 2-oxovalerate aldolase |  |
| M-Z18AGL000937 | mhpF | acetaldehyde dehydrogenase |  |
| M-Z18AGL008142 | E1.1.1.90 | aryl-alcohol dehydrogenase |  |
| M-Z18AGL000854 | dhaA | haloalkane dehalogenase | Chlorocyclohexane and chlorobenzene degradation |
| M-Z18AGL008478 | E3.8.1.2 | 2-haloacid dehalogenase |  |
| M-Z18AGL000758 | linC | 2,5-dichloro-2,5-cyclohexadiene-1,4-diol dehydrogenase |  |
| M-Z18AGL007917 | E3.1.2.23 | 4-hydroxybenzoyl-CoA thioesterase | Benzoate degradation |
| M-Z18AGL008298 | pcaB | 3-carboxy-cis,cis-muconate cycloisomerase |  |
| M-Z18AGL000938 | mhpD | 2-keto-4-pentenoate hydratase |  |
| M-Z18AGL000936 | mhpE | 4-hydroxy 2-oxovalerate aldolase |  |
| M-Z18AGL000937 | mhpE | acetaldehyde dehydrogenase |  |
| M-Z18AGL000586 | ligK | 4-hydroxy-4-methyl-2-oxoglutarate aldolase |  |
| M-Z18AGL008643 | DDC | aromatic-L-amino-acid decarboxylase | Tryptophan metabolism |
| M-Z18AGL008353 | pcaB | catalase-peroxidase |  |
| M-Z18AGL008462 | ACMSD | aminocarboxymuconate-semialdehyde decarboxylase |  |
| M-Z18AGL005943 | pulA | pullulanase | Starch and sucrose metabolism |
| M-Z18AGL000918 | sapM | phosphatidylinositol-3-phosphatase | Inositol phosphate metabolism |
| M-Z18AGL008389 | lldD | L-lactate dehydrogenase | Pyruvate metabolism |
| M-Z18AGL000937 | mhpF | acetaldehyde dehydrogenase |  |
| M-Z18AGL008142 | E1.1.1.90 | aryl-alcohol dehydrogenase | Xylene degradation |
| M-Z18AGL000938 | mhpD | 2-keto-4-pentenoate hydratase |  |
| M-Z18AGL000936 | mhpE | 4-hydroxy 2-oxovalerate aldolase |  |
| M-Z18AGL000937 | mhpF | acetaldehyde dehydrogenase |  |
| M-Z18AGL008142 | E1.1.1.90 | aryl-alcohol dehydrogenase | Phenylalanine metabolism  Chlorocyclohexane and chlorobenzene degradation |
| M-Z18AGL000854 | dhaA | haloalkane dehalogenase |  |
| M-Z18AGL008478 | E3.8.1.2 | 2-haloacid dehalogenase |  |
| M-Z18AGL000249 | mdlC | benzoylformate decarboxylase | Aminobenzoate degradation  Styrene degradation  Butanoate metabolism |
| M-Z18AGL000942 | gctA | glutaconate CoA-transferase |  |
| M-Z18AGL000709 | butB | (R,R)-butanediol dehydrogenase |  |
| M-Z18AGL000024 | bdh | 3-hydroxybutyrate dehydrogenase |  |
| M-Z18AGL008130 | ttuC | tartrate dehydrogenase |  |
| M-Z18AGL000937 | mhpF | acetaldehyde dehydrogenase |  |
| M-Z18AGL000942 | gctA | glutaconate CoA-transferase, subunit A |  |
| M-Z18AGL000761 | phbB | acetoacetyl-CoA reductase |  |
| M-Z18AGL008294 | scoA | 3-oxoacid CoA-transferase subunit A |  |
| M-Z18AGL000586 | ligK | 4-hydroxy-4-methyl-2-oxoglutarate aldolase | C5-Branched dibasic acid metabolism |
| M-Z18AGL000916 | ict-P | itaconate CoA-transferase |  |
| M-Z18AGL000687 | comB | 2-phosphosulfolactate phosphatase | Methane metabolism |
| M-Z18AGL008430 | mfnB | (5-formylfuran-3-yl)methyl phosphate synthase |  |
| M-Z18AGL008377 | frhB | coenzyme F420 hydrogenase subunit beta |  |
| M-Z18AGL000192 | ssuE | FMN reductase | Riboflavin metabolism  Limonene and pinene degradation  Nitrogen metabolism |
| M-Z18AGL000192 | ssuE | FMN reductase |  |
| M-Z18AGL000376 | narG, narZ, nxrA | nitrate reductase / nitrite oxidoreductase, alpha subunit |  |
| M-Z18AGL008242 | cynS | cyanate lyase |  |
| M-Z18AGL007865 | SUOX | sulfite oxidase | Sulfur metabolism  Caprolactam degradation  Steroid degradation |
| M-Z18AGL008571 | mlhB | epsilon-lactone hydrolase |  |
| M-Z18AGL000180 | tesI | 3-oxo-5alpha-steroid 4-dehydrogenase |  |
| M-Z18AGL000926 | kstD | 3-oxosteroid 1-dehydrogenase |  |
| M-Z18AGL000933 | kshB | 3-ketosteroid 9alpha-monooxygenase subunit B |  |
| M-Z18AGL000929 | hsaC | 3,4-dihydroxy-9,10-secoandrosta-1,3,5(10)-triene-9,17-dione 4,5-dioxygenase |  |
| M-Z18AGL000930 | hsaD | 4,5:9,10-diseco-3-hydroxy-5,9,17-trioxoandrosta-1(10),2-diene-4-oate hydrolase |  |
| M-Z18AGL008562 | rifA | rifamycin polyketide synthase modules 1, 2 and 3 | Biosynthesis of ansamycins |
| M-Z18AGL000287 | asm16 | methoxymalonate biosynthesis protein |  |
| M-Z18AGL000289 | asm14 | methoxymalonate biosynthesis acyl carrier protein |  |
| M-Z18AGL008220 | asm10 | N-methyltransferase |  |
| M-Z18AGL008346 | asm25 | N-glycosyltransferase |  |
| M-Z18AGL001785 | eryK | erythromycin 12 hydroxylase | Type I polyketide structures |
| M-Z18AGL007293 | nidA4_5 | niddamycin polyketide synthase 4/5 |  |
| M-Z18AGL000212 | aveF | C-5 ketoreductase |  |
| M-Z18AGL008562 | rifA | rifamycin polyketide synthase modules 1, 2 and 3 |  |
| M-Z18AGL000287 | asm16 | methoxymalonate biosynthesis protein |  |
| M-Z18AGL000289 | asm14 | methoxymalonate biosynthesis acyl carrier protein |  |
| M-Z18AGL008220 | asm10 | N-methyltransferase |  |
| M-Z18AGL000417 | amphA | polyene macrolide polyketide synthase, loading module |  |
| M-Z18AGL000416 | amphB | polyene macrolide polyketide synthase, A-type KR domains |  |
| M-Z18AGL000414 | amphC | polyene macrolide polyketide synthase |  |
| M-Z18AGL000424 | amphK | polyene macrolide polyketide synthase |  |
| M-Z18AGL000425 | amphI | polyene macrolide polyketide synthase, KS-AT-KR-ACP domains |  |
| M-Z18AGL008282 | CYP140A7 | cytochrome P450 hydroxylase |  |
| M-Z18AGL000744 | pchB | isochorismate pyruvate lyase | Biosynthesis of siderophore group nonribosomal |
| M-Z18AGL000271 | entE | 2,3-dihydroxybenzoate-AMP ligase |  |
| M-Z18AGL008209 | nocF | 4-hydroxymandelate synthase | Biosynthesis of vancomycin group antibiotics |
| M-Z18AGL008207 | nocN | 4-hydroxymandelate oxidase |  |
| M-Z18AGL008205 | nocG | (S)-3,5-dihydroxyphenylglycine transaminase |  |
| M-Z18AGL007972 | calG2 | calicheamicin 4-deoxy-4-thio-alpha-D-digitoxosyltransferase | Biosynthesis of enediyne antibiotics |
| M-Z18AGL000376 | narG, narZ, nxrA | nitrate reductase / nitrite oxidoreductase, alpha subunit | Two-component system |
| M-Z18AGL000377 | narH, narY, nxrB | nitrate reductase / nitrite oxidoreductase, beta subunit |  |
| M-Z18AGL000378 | narJ, narW | nitrate reductase molybdenum cofactor assembly chaperone NarJ/NarW |  |
| M-Z18AGL000379 | narI, narV | nitrate reductase gamma subunit |  |
| M-Z18AGL008680 | phzF | trans-2,3-dihydro-3-hydroxyanthranilate isomerase | Quorum sensing |
| M-Z18AGL008343 | rnhA | ribonuclease HI | DNA replication |
| - | - | - | phosphatidylinositol signaling system |
| - | - | - | protein process in endoplasmic reticulum |
| M-Z18AGL008230 | htpG | molecular chaperone HtpG | NOD-like receptor signaling pathway |
| M-Z18AGL008230 | htpG | molecular chaperone HtpG | Plant-pathogen interaction |

-represent deletion metabolic pathway
